# The cardiovascular risk of celecoxib for knee osteoarthritis: A protocol for systematic review and meta-analysis

**DOI:** 10.1097/MD.0000000000019976

**Published:** 2020-05-01

**Authors:** Shirui Cheng, Ming Xin, Jun Zhou, Ying Cheng, Guixing Xu, Yuanfang Zhou, Zhengjie Li, Fanrong Liang

**Affiliations:** a The Acupuncture and Tuina School/The 3rd Teaching Hospital, Chengdu University of Traditional Chinese Medicine; b The Rehabilitation Department, Chengdu Fifth People's Hospital, Chengdu, Sichuan, China; c The Cheng Clinic Limited, London, England.

**Keywords:** cardiovascular risk, celecoxib, knee osteoarthritis, meta-analysis, protocol, systematic review

## Abstract

**Objective:**

The aim of this systematic review and meta-analysis is to assess the cardiovascular (CV) risk of celecoxib on knee osteoarthritis (KOA) patients compared with the risk in those prescribed other non-selective non-steroidal anti-inflammatory drugs (NSAIDs), no intervention or placebo-controlled patients.

**Methods:**

The following databases will be searched: MEDLINE, EMBASE, the Cochrane Library, Web of Science, Chinese Biomedical Literature Database, Chinese Nation Knowledge Infrastructure, Wanfang Database, and the Chongqing VIP from inception to April 1, 2020. All randomized controlled trials (RCTs) of celecoxib that presented data on serious cardiovascular events among KOA patients will be included. Study selection, data extraction, quality assessment, and assessment of risk bias will be performed by 2 reviewers independently. Odds ratios and correlative 95% confidence intervals will be calculated to present the association between the celecoxib and CV risk using Review Manager version 5.3 when there is sufficient available data.

**Ethics and dissemination:**

This review does not require ethical approval. The results of this review may be published in a peer-reviewed journal or disseminated at relevant conferences.

**PROSPERO registration number:**

CRD42020166721.

## Abstract 2

Supplemental Digital Content is available in the text

## 1 Introduction

Knee osteoarthritis (KOA) is a complex chronic arthritis, characterized by focal loss of articular cartilage, marginal and central new bone formation.^[1,2]^ Patients with KOA often suffer from joint pain, joint swelling, knee dysfunction, and even joint deformity.^[2]^ Approximately 35% of women and men aged 60 years and above have radiographic KOA^[3]^ and nearly half of these people have symptoms,^[2,4]^ affecting 9 million individuals in the United States.^[5]^ KOA, combined with hip osteoarthritis, are the 11th leading cause of global disability, accounting for 2.2% of total years lived with disability.^[6]^ Physical disability arising from pain and loss of functional capacity reduces quality of life and increases the risk of further morbidity and mortality.^[1,7]^

Current treatments are primarily prescribed to relieve pain because the first consultation of a physician usually is due to painful knee joints. The main interventions to treat KOA including patient education, pharmacological therapy, and surgery.^[1,8–10]^ Among them, the nonsteroidal anti-inflammatory drugs (NSAIDs) are the most widely used analgesics to treat KOA in clinic.^[11]^ Traditional NASAIDs have significant gastrointestinal toxicity because of the inhibition of cyclooxygenase 1 (COX-1), which predominates in the stomach. Thus, selective COX-2 inhibitors (coxibs) have been developed to reduce the adverse gastrointestinal effects. However, long-term use of coxibs are associated with an increased risk of acute cardiovascular (CV) events, which has brought about the withdrawal of rofecoxib and other coxibs from the market.^[12–14]^

Celecoxib is the first specific inhibitor of COX-2.^[15]^ It is equipotent in terms of pain management in OA and rheumatoid arthritis when compared to nonselective NSAIDs,^[16,17]^ and has better tolerability for both upper and lower gastrointestinal tract.^[18,19]^ Celecoxib was approved by the US Food and Drug Administration for adult patients with OA,^[15]^ while it is contraindicated in patients with established cardiovascular disease in Europe.^[20,21]^ Caldwell et al. investigated the CV risk of celecoxib therapy during the patients with OA, RA, Alzheimer's disease, and with high risk of colorectal adenoma, found that celecoxib had an increased risk of myocardial infarction.^[22]^ While in a review about the efficacy and safety of celecoxib on KOA, Puljak et al found the cardiovascular events in celecoxib group had no statistical significance when compared with placebo group, but this result was inconclusive according to the low quality evidence.^[23]^ Data on the cardiovascular risk of celecoxib remain in conflict.^[24]^

To clarify this issue, we will undertake a systematic review and meta-analysis of double-blind, randomized, controlled studies of celecoxib that presented data on serious cardiovascular events among KOA patients. We will compare the risk of CV events in KOA patients prescribed celecoxib with the risk in those prescribed other non-selective NSAIDs, no intervention or placebo-controlled patients.

## 2 Methods and design

### 2.1 Inclusion criteria

#### 2.1.1 Type of studies

Randomized controlled trials (RCT) reported in English or Chinese will be included. Other study design will be excluded. There will be no restriction on publication date.

#### 2.1.2 Type of participants

At least 75% of participants with clinically or radiologically confirmed primary KOA. There will be no restrictions on age, gender, race, or nation. The diagnosis of KOA will be based on valid clinical and radiographic findings in accordance with the American College of Rheumatology criteria.

#### 2.1.3 Type of interventions

Oral celecoxib versus no intervention, placebo or other nonselective NSAIDs. Besides, celecoxib plus other interventions will also be included. There will be no restrictions on the dose, frequency of taking celecoxib.

#### 2.1.4 Type of comparators

The control group with no interventions, placebo or other nonselective NSAIDs will be included. There will be no restrictions on the dose, frequency of comparators.

#### 2.1.5 Outcome measurements

Primary outcome will be the number of participants with CV events, including myocardial infarction, cardiovascular mortality, heart failure, and unstable angina. The main time endpoint will be the occurrence of any cardiovascular events. And reporting of cardiovascular outcomes will rely on study-specific outcome definitions. Secondary outcomes will be the number of other cardiovascular disease events, including atrial fibrillation, arrhythmias, arterial hypertension; the number of cerebrovascular events, including ischemic or hemorrhagic stroke events. The time endpoint will be the occurrence of above events.

### 2.2 Exclusion criteria

Articles will be excluded if they meet one of the following criteria: (1) be non-RCTs, quasi-RCTs, crossover trials, case report, animal studies, expert's experience, and conference articles; (2) not providing any cardiovascular outcomes; (3) participants were not free of cardiovascular disease at baseline.

### 2.3 Search strategy

#### 2.3.1 Electronic searches

From the inception to April 1, 2020, the following databases will be searched: MEDLINE, EMBASE, the Cochrane Library, Web of Science, Chinese Biomedical Medical Database, Chinese Nation Knowledge Infrastructure, Wanfang Database, and the Chongqing VIP. The searching strategy of MEDLINE is presented in Supplemental Digital Content (Appendix 1). This search strategy will be modified to be suitable for other electronic databases.

#### 2.3.2 Searching other resources

Unpublished or ongoing trial data will also be searched from the following clinical trial registries: The National Institutes of Health (NIH) clinical registry Clinical Trials, the Chinese clinical registry, the Australian New Zealand Clinical Trials Registry, and the International Clinical Trials Registry Platform (ICTRP). Ambiguous literatures and reference lists of identified publications will be checked manually.

### 2.4 Data collection

#### 2.4.1 Studies selection

**Figure 1:**
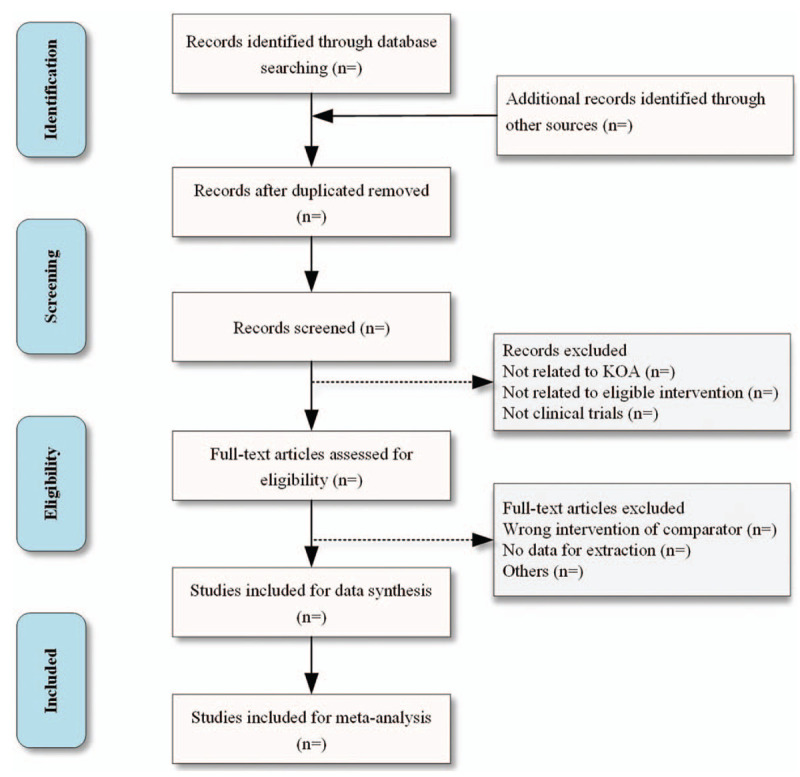
Flow diagram of study selection process.

All the studies of electronic searches and other sources will be imported to Endnote version X9 software. The duplicated studies will be filtered. The titles and abstracts of potentially qualified studies will be screened by two reviewers (SC and MX) independently. And the studies not meeting the inclusion criteria will be excluded. The full text will be further screened if the studies cannot be estimated according to the titles and abstracts. After screening, two reviewers will cross-check the included studies. The inconsistent opinions between the two reviewers will be resolved through discussion. If no agreement is reached, a third reviewer (JZ) will be consulted. Details of the selection procedure for studies are shown in a Preferred Reporting Item for Systematic review and Meta-analysis protocol (PRISMA-P) flow chart (Fig. 1).

#### 2.4.2 Data extraction

The following information from the included studies will be extracted by two reviewers (SC and MX) using a standard form:

(1) General information: surname of first author, year of publication, statistical approaches, publication source, country, funding supports;
(2) Participants: sex, the mean age of participants, illness duration, ethnicity, education;
(3) Study characteristics: design, sample size, diagnostic criteria, method of randomization, blinding, interventions and controls, method of analysis, outcome measures, time of follow-up;
(4) Results: number of CV events.

After extraction, two reviewers will cross-check. The disagreement between the two reviewers will be solved by discussion. The extraction data will be listed in Excel 2016, and JZ will check the entered data to ensure the consistency and correct data entry errors.

### 2.5 Risk of bias assessment

The risk of bias of the included trials will be evaluated by two reviewers (SC and JZ) independently using the Cochrane risk of bias tool (http://www.cochrane-handbook.org.). The following six items will be assessed, including: random sequence generation, allocation concealment, blind subjects and therapists, blind assessors, incomplete outcome data, selective reporting, and other bias when required.^[25]^ The risk of bias in each item is rated as high, low risk, or unclear of bias. The rating results will be cross-checked and disagreements will be resolved through discussion or the third reviewer (MX).

### 2.6 Dealing with missing data

If the data of the primary studies is missing, the authors will be contact for the information. If the missing data cannot be obtained, the studies will only be included for narrative analysis.

### 2.7 Quality assessment

The quality of evidence of outcomes will be assessed by two reviewers (SC and JZ) independently according to the Grading of Recommendations Assessment, Development and Evaluation (GRADE) system. The GRADE system includes five items: the risk of bias, inconsistency, indirectness, imprecision, and publication bias.^[26,27]^ The quality of evidence will be rated as “high”, “moderate”, “low” or “very low”.

### 2.8 Data analysis

#### 2.8.1 Data synthesis

The odds ratios (ORs) and correlative 95% confidence intervals (CIs) will be calculated to present the association between the celecoxib and CV risk using Review Manager version 5.3 (RevMan V5.3, the Nordic Cochrane Centre, Copenhagen, Denmark). If excessive statistical heterogeneity does not exist, we will pool data across studies using fixed-effects model for meta-analysis. When statistical heterogeneity exists, a random-effects model will be used for meta-analysis. Besides, a narrative and qualitative summary will also be provided. A two-side p value less than 0.05 in the Z-test will be regarded as significant.

#### 2.8.2 Assessment of heterogeneity

The heterogeneity will be analyzed through chi-squared (*X*^2^) test by using RevMan V5.3 according to the Cochrane Handbook. P value of less than 0.10 will be considered significant. Moreover, we will calculate the I^2^ value on the meta-analysis to quantify the impact of the statistical heterogeneity. The I^2^ value is defined into 4 categories according to the Cochrane Handbook: 0% to 40%, might not be important; 30% to 60%, represents moderate heterogeneity; 50% to 90%, suggests substantial heterogeneity; 75% to 100%, indicates considerable heterogeneity. The potential cause of the significant heterogeneity will be further explored using meta-regression analysis.

#### 2.8.3 Subgroup analysis

In meta analyses where we find substantial heterogeneity, we will perform prespecified subgroup analyses on the dose of celecoxib, age, sex, control interventions, treatment frequency, as data allows. Where we identify unexplained substantial heterogeneity, we will not pool results into an overall effect estimate but rather present the individual effect sizes per study for the specific outcome.

#### 2.8.4 Sensitivity analysis

In order to verify the stability of the primary outcomes, sensitivity analysis will be performed according to the sample size, study design, methodological quality, and the effect of missing data of the included studies.

#### 2.8.5 Assessment of publication biases

Funnel plots will be performed to assess the reporting bias when more than 10 trials are included. If the funnel plots are asymmetric, we will try to interpret the funnel plots asymmetry.

## 3 Results reporting and presentation

The results of this systematic review and meta-analysis will be reported according to the Consolidation of Standard for Reporting Trials guidelines (CONSORT),^[28]^ the recommendations described in PRISMA statement, and the Cochrane Handbook for Intervention Reviews.

There are some limitations in this review. This study tries to evaluate the cardiovascular risk of celecoxib on the treatment of knee osteoarthritis by both qualitative and quantitative analysis. Some RCTs did not report the CV events, and we might not acquire the related data. Because of the barrier of language, only trials published in English or Chinese will be included.

## Author contributions

**Conceptualization:** S Cheng, Z Li, F Liang.

**Data curation:** S Cheng, J Zhou.

**Methodology:** S Cheng, M Xin, J Zhou.

**Project administration:** Y Cheng.

**Writing – original draft:** S Cheng, M Xin, J Zhou.

**Writing – review & editing:** G Xu, Y Z, Z Li, F Liang.

## Supplementary Material

Supplemental Digital Content
