# Supplementary material for: The cardiovascular risk of celecoxib for knee osteoarthritis: A protocol for systematic review and meta-analysis
Source: Medicine (Baltimore). 2020 May 1;99(18):e19976. doi: 10.1097/MD.0000000000019976 (PMC7440337; doi:10.1097/MD.0000000000019976)
Supplement: Supplemental Digital Content [file medi-99-e19976-s001.docx]

**MEDLINE (OVID)**

1 (cyclooxygenase-2 or cyclooxygenase 2 or cyclooxygenase-II or cyclooxygenaseII).ti,ab.

2 (cyclo oxygenase-2 or cyclo oxygenase 2 or cyclo oxygenase-II or cyclo oxygenaseII).ti,ab.

3 (cox-2 or cox2 or cox-II or coxII).ti,ab.

4 (celecoxib or celebrex or SC-58635).af

5 exp Cyclooxygenase inhibitors/

6 or/1-5

7 exp osteoarthritis/

8 osteoarthr$.tw.

9 (degenerative adj2 arthritis).tw.

10 arthrosis.tw.

11 or/7-10

12 Knee/

13 exp Knee Joint/

14 knee$.tw.

15 or/12-14

16 11 and 15

17 clinical trial.pt.
18 randomized.ab. or randomized controlled trial.pt. or controlled clinical trial.pt.
19 placebo.ab.
20 exp Clinical Trial/
21 randomly.ab.
22 trial.ti.
23 or/17-22
24 Animals/
25 Humans/
26 24 not (24 and 25)
27 23 not 26

28 6 and 16 and 27
